# Plant-Derived Polyphenols to Prevent and Treat Oral Mucositis Induced by Chemo- and Radiotherapy in Head and Neck Cancers Management

**DOI:** 10.3390/cancers16020260

**Published:** 2024-01-06

**Authors:** Elena Belfiore, Giulia Di Prima, Giuseppe Angellotti, Vera Panzarella, Viviana De Caro

**Affiliations:** 1 Department of Surgical, Oncological and Oral Sciences, University of Palermo, Via L. Giuffrè 5, 90127 Palermo, Italy; elena.belfiore@unipa.it (E.B.); vera.panzarella@unipa.it (V.P.); 2 Department of Biological, Chemical and Pharmaceutical Sciences and Technologies, University of Palermo, Via Archirafi 32, 90123 Palermo, Italy; giulia.diprima@unipa.it; 3 Institute of Nanostructured Materials, National Research Council, Via U. La Malfa 153, 90146 Palermo, Italy; giuseppe.angellotti@cnr.it

**Keywords:** oral mucositis, polyphenols, head and neck cancers, phytocomplex, curcuminoids, flavonoids, quercetin, apigenin, epicatechin, hesperidin

## Abstract

**Simple Summary:**

Oral Mucositis, a debilitating side effect of radio and chemotherapy for head and neck cancers, involves inflammation and ulceration of the mucous membranes in the oral cavity. This condition often leads to severe pain, difficulty in eating, and compromised quality of life for cancer patients. The use of natural compounds such as polyphenols has shown promise in preventing and alleviating Oral Mucositis as they possess anti-inflammatory, antioxidant, and healing properties, capable of mitigating the adverse effects of chemo and radiotherapy on the oral mucosa. The recent literature suggests that integrating these natural compounds into treatment regimens may help manage Oral Mucositis, offering a potential adjuvant therapy to improve a patient’s quality of life and overall well-being during cancer treatment.

**Abstract:**

Oral Mucositis (OM) is the most common side effect due to chemotherapy and radiotherapy, which are the conventional treatment options for head and neck cancers. OM is a severe inflammatory condition characterized by multifactorial etiopathogenesis. It further negatively affects patients’ quality of life by severe impairment of normal oral functions. Consequently, it is mandatory to identify new effective therapeutic approaches to both prevent and treat OM while also avoiding any recurrence. Polyphenols recently attracted the interest of the scientific community due to their low toxicity and wide range of biological activities making them ideal candidates for several applications in the odontostomatological field, particularly against OM. This review collects the in vivo studies and the clinical trials conducted over the past 13 years evaluating the preventive and curative effects of several polyphenolic compounds towards chemo- and radiotherapy-induced OM, both when administered alone or as a plant-extracted phytocomplex. The literature fully confirms the usefulness of these molecules, thus opening the possibility of their clinical application. However, polyphenol limitations (e.g., unfavourable physicochemical properties and susceptibility to degradation) have emerged. Consequently, the interest of the scientific community should be focused on developing innovative delivery systems able to stabilize polyphenols, thus facilitating topical administration and maximizing their efficacy.

## 1. Introduction

Head and neck cancers are common tumours, representing approximately 10% of all malignancies in men and 5% in women [1]. They originate in the upper aero-digestive tract at various levels including the larynx, upper trachea, pharynx, and oral and nasal cavity [2]. The onset of these diseases is currently upward and seems to be related to some diffuse risk factors such as consumption of alcohol and tobacco (separately or in combination) and virus infections (e.g., Human Papilloma Virus—HPV) [1,3]. Among the head and neck cancers, oral cancers are remarkably relevant for human health and about 90% of them belong to the squamous cell carcinoma type. In particular, Oral Squamous Cell Carcinoma (OSCC) represents the most frequent malignant tumour affecting the oral mucosal epithelium, with a higher incidence in the male population [4], although a share of OSCC appears to involve an increasingly younger population (under 40). Despite the progress in therapy, the mortality of patients with OSCC has remained steadily high during the last 20 years as compared to other cancers [5]. Its early detection and treatment are still crucial to improve the prognosis, and, in this regard, a combination of multiple therapeutic approaches is frequently recommended to remove cancer and prevent any recurrence [6]. Conventional therapies include surgery, radiotherapy, and chemotherapy; however, these are characterized by several side effects and low patient compliance. Therefore, other more specific treatments such as localized and personalized therapy (which target some genes in cancer cells), administration of natural molecules as adjuvant and chemopreventive agents [7,8], as well as immunotherapy with monoclonal antibodies could be employed [9]. The routine recommendation is surgical management of the primary tumour followed by post-operative radiotherapy or chemoradiotherapy depending on the presence of intermediate/high risk. However, radio- and chemotherapy currently remain the most common therapeutic strategies [10]. Chemotherapy is generally based on drugs such as 5-Fluorouracil (5-FU), Cisplatin, Cetuximab, and Taxanes which being non-selective drugs [11,12] are characterized by several side effects such as gastrointestinal disorders, immune and hematopoietic deficit with consequent infections, inflammation and mucositis in the entire gastrointestinal tract including the oral cavity. On the other hand, head and neck radiotherapy shows unpleased local effects such as severe hyposalivation/xerostomia due to impairment of normal salivary gland function, damage of the oral mucosa, and dermatitis in the overlying epithelium [13]. Major damage to the oral cavity occurs if chemotherapy is associated with radiotherapy as their synergistic effect in the reduction of the epithelial cell turnover leads to thinning of the mucosa itself causing a loss of integrity. Therefore, a compromised function of the epithelium together with a strong inflammation process, oxidative environment, and high susceptibility to bacterial infections often originate in a complex clinical scenario defined as Oral Mucositis (OM) [14]. Considering that oral cancer itself is a disabling disease with a low survival rate, the onset of OM during treatments could further compromise the patient’s overall conditions, lowering their quality of life due to the severe repercussions on nutrition and other mouth functions (as speaking, digesting, or even simply opening the mouth).

## 2. Oral Mucositis (OM)

OM is a severe acute inflammation affecting the oral mucosa characterized by tissue swelling, ulceration, and erythema [15]. OM develops in around 40–60% of patients with head and neck cancer who receive standard radiotherapy and chemoradiotherapy [16]. OM is described as a painful condition generally resulting in hard discomfort and a negative impact in terms of patients’ quality of life. Depending on the cancer treatment used, it is characterized by a different onset. The common signs that characterize OM are erythema, erosions, and ulcerations of the tissues, which cause a painful condition that can be severe, depending on the size and localization of the damage to the mucous membrane [17,18]. This condition can be further worsened by bacterial infections [4]. In this regard, the World Health Organization (WHO) developed a grading system for the OM severity, ranging from 0 (no OM) to 4 (highly severe OM), which is used to assess its clinical features such as symptoms (e.g., pain), signs (e.g., erythema and ulceration), and oral function (e.g., swallowing ability). This scale also evaluates the severity of clinical manifestations which depends on several factors, such as the type of treatment, the dose, and such individual variables (response to treatment, age, diet, oral hygiene, tumour type, and genetic factors) [19]. Although the onset of OM is the result of different mechanisms that may occur simultaneously, it is possible to identify a common sequence of events that characterizes the pathogenesis of both chemo- and radiotherapy-induced OM. The model described by Sonis in 2004 [20] is useful for the recognition of this sequence of five events:

- Inflammatory/vascular phase: It is induced by chemotherapy and/or radiotherapy, which induce cytotoxicity in normal cells by directly damaging the DNA and leading to excessive ROS generation. This phenomenon acts as a trigger for the inflammatory process activating different signalling pathways such as proinflammatory cytokines (e.g., IL1 β, IL6, and TNF-α) and prostaglandins [21,22].
- Activation of transcription factors such as nuclear factor-κ B (NF-κB) and NF-E2-related factor 2 (Nrf2) which can be directly activated by the chemotherapeutic agents, e.g., 5-FU activates the NF-κB, thereby upregulating the genes encoding pro-inflammatory cytokines such as Tumour Necrosis Factor α (TNF-α), interleukin 1β (IL-1β), and IL-6, cyclooxygenase 2 (COX-2), and high-mobility group box 1 protein (HMGB1) or radiation, and indirectly through the ROS release, producing inflammatory mediators, which increase the tissue damage stimulating angiogenesis and vascular permeability [23].
- Up-regulation and signal amplification stage, leading to loss of the epithelium integrity and, hence, ulcer formation (beginning of OM evolution).
- Rich inflammatory infiltrate stage, containing macrophages, neutrophils, and mastocytes [24]. In addition, lesions are strongly subjected to bacterial colonization, which contributes to stimulating the innate immune system, thereby increasing the inflammatory response.
- The final healing phase, characterized by the proliferating and differentiating epithelial cells, leading to the restoration of the integrity of altered mucosa.

Almost 20–40% of cancer patients undergoing conventional chemotherapy manifest this condition (CT-OM) within 4–7 days of treatment, and the clinical signs and symptoms continue for 1 to 2 weeks after.

In head and neck cancer patients, immunotherapy is amongst the most promising strategies. Moreover, the prevalence of related OM seems to be lower compared to traditional chemotherapeutics (methotrexate, cisplatin) [25].

However, OM represents a potential side effect of both targeted therapy and immunotherapy (mammalian target of rapamycin inhibitor-associated stomatitis (mIAS) and immunotherapy-related adverse events (irAEs)) with both severity and clinical presentation depending on the agent used [26]. When combined with conventional chemotherapy, these therapies may increase the risk and severity of mucosal involvement, with a combined presentation of both superficial and deeper, classic Oral Mucositis ulcers [27].

Moreover, Amy et al. reported severe chronic OM associated with pembrolizumab immunotherapy lasting for months even after the drug was stopped and representing the major cause of suffering and eating difficulties for cancer patients [28].

Also, radiation therapy for head and neck cancer causes OM; in this case, side effect onset is delayed, but the duration is longer (between 2 and 6 weeks). Radiation therapy-induced OM (RT-OM) depends on the dose administered, the volume of the tissue treated, and the type of radiation used. The sequelae of CT/RT-OM, including pain, odyno/dysphagia, dysgeusia, decreased oral intake, and local/systemic infection, often require treatment delays, interruptions, and discontinuations which have negative impacts not only on the quality of life but also on tumour control and survival [29].

Currently, the clinical management of OM is mainly aimed at alleviating symptoms, and it is therefore based on the use of conventional drugs suitable for treating pain, inflammation, and infection such as topical anaesthetics (e.g., lidocaine), systemic opioid analgesics (e.g., morphine and fentanyl), and topical and systemic antibiotics and antifungals, which are generally recommended for the prevention of infection [30,31]. Treatment of oral mucosal irAEs is generally carried out with high-potency topical steroids, or systemic steroid/immunosuppressive agents [26].

However, preventing and/or treating any recurrence remains quite complex and often requires multiple approaches which can cause multiple side effects. Therefore, the identification of alternative ways to prevent and treat CT and RT–OM is necessary, especially considering the new targets recently discovered in the pathogenesis of mucosal damage [32].

As the abnormal release of Reactive Oxygen Species (ROS) and the massive inflammatory phase are the main causes of the onset and recurrence of OM, the use of natural antioxidants and anti-inflammatory compounds could be a wise strategy. Among these, polyphenols have recently emerged [33].

### Polyphenols in the Prevention and Management of OM

Naturally, plants produce a wide variety of molecules in response to various environmental stimuli (e.g., microbial infection, temperature, etc.). The same compounds, due to their natural actions, could also show interesting properties in humans, useful in the prevention and treatment of several disorders [34,35,36]. Among the wide variety of plant-derived bioactive molecules, polyphenols represent a class of secondary metabolites that are widely distributed in the plant kingdom and are generally synthesized as a defense against such environmental stresses and pathogens [37]. Tea, cocoa powder, grapes, and spices are just a few examples of rich natural sources of polyphenols, but they are also found in edible plants and natural products such as propolis and honey [38].

**Figure 1:**
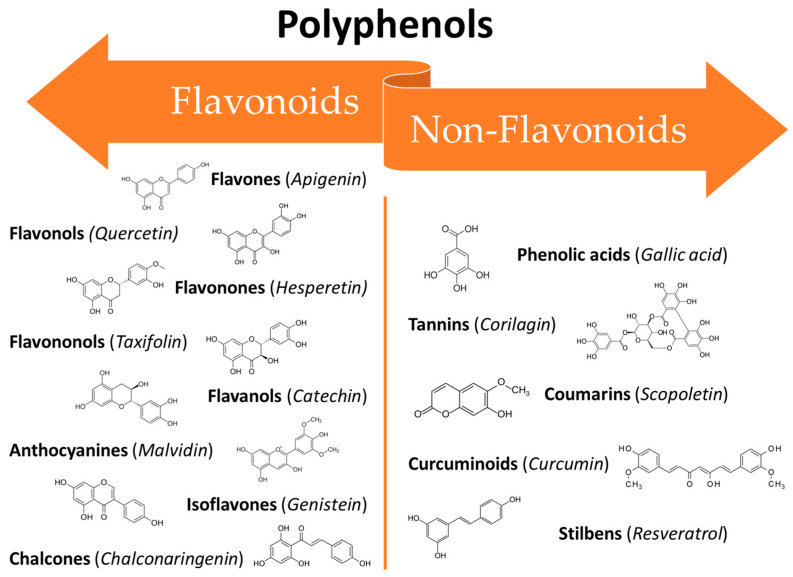
Classification of polyphenols: main classes and subclasses with some representative molecules.

The large class of polyphenols comprise a broad group of phytochemicals that includes hundreds of natural molecules which are divided into different subclasses according to their main chemical structure. These compounds range from small molecules such as phenolic acids or stilbenes to larger molecules such as tannins [39]. The chemical characteristic that units all of the polyphenols are a phenolic structure based on phenyl ring(s) as a core which presents one or more hydroxyl substituents, essential for the radical scavenging activity (due to electron delocalization and stabilization of the formed radical) [37]. According to their structure polyphenols can be classified into two main groups: flavonoids and non-flavonoids (Figure 1). Flavonoids possess two phenyl rings fused with a central heterocyclic ring containing oxygen [40]. They can be further subdivided into flavonols, flavones, flavanones, flavanols isoflavones, anthocyanidins, and chalcones.

On the other hand, the non-flavonoids can be divided into different subclasses such as phenolic acids, tannins, coumarins, curcuminoids, and stilbenes [41]. In recent years, polyphenols have gained the attention of researchers all around the world thanks to their broad spectrum of bioactive properties, which may be useful in improving human health, as well as their low side effects. They can interact with different cellular pathways, enzymatic mechanisms, and hormones controlling gene expression [42]. Thus, the mechanisms involved in their activity are complex and often still controversial. Their wide range of biological activities includes antioxidant [38], antifungal [43,44], anti-inflammatory [45], anti-aging [46], osteogenic [47], chemopreventive [48], and antitumoural [49] properties which makes them potentially useful in several fields ranging from pharmaceuticals to cosmetics [50]. Several in vitro studies, in vivo studies, and clinical trials have been conducted to assess the efficacy of polyphenols against the oral mucosa damage that occurs as a side effect of anticancer therapies. The broad-spectrum activities of polyphenols make them ideal candidates for clinical use in many areas of medicine. However, their use is still limited due to their unfavourable physicochemical properties such as low water solubility, instability at high temperatures and alkaline pH, as well as a massive first-pass effect after oral administration, resulting in extremely low bioavailability [51]. Recently, researchers have proposed several innovative solutions to mitigate these drawbacks in order to benefit from the therapeutic effects of these potent molecules. Some of the most recent innovations are related to new techniques for their extraction and manipulation and the design of innovative drug delivery platforms and systems suitable for minimizing the degradation phenomena and improving bioavailability [51]. Based on these considerations, the aim of this work is to provide an overview of the in vivo studies and clinical trials collected in recent years that enlighten the usefulness of polyphenols in dentistry for the prevention and treatment of radio- and chemotherapy-induced OM.

## 3. Methodology

The papers collected in this review were selected according to the following inclusion criteria: (i) manuscripts published in the English language within the last 13 years in peer-reviewed scientific journals of relevance in the oncology field; (ii) in vivo studies conducted on animal models in which OM was clinically induced by radio- or chemotherapy and treated with polyphenols before or together with the anticancer treatments; and (iii) phase 1 and phase 3 clinical trials including patients affected by head and neck cancers with OM induced by radiotherapy, chemotherapy or a combination thereof. In contrast, the exclusion criteria were the following: (i) studies published in a native language different from English and/or in a journal without a peer-review process; (ii) in vivo and clinical studies that did not report the ethical guidelines; (iii) in vitro studies; and (iv) clinical trials or in vivo studies published before 2010. Three databases were used for papers’ screening: PubMed, Google Scholar, and Scopus.

## 4. Curcuminoids

Curcuminoids are the main active components of the rhizomes of *Curcuma longa* L., an herb widely used as a traditional remedy in China and Southeast Asia. The term “curcuminoids” indicates a mixture of compounds such as curcumin, Dimethoxy curcumin, and Bis-dimethoxy curcumin. Furthermore, there is also a fourth molecule called Cyclocurcumin, which was initially identified as curcuminoid but was later considered only to be a structural isomer of curcumin [52]. Curcuminoids are composed of a core structure having two aromatic benzene methoxy rings linked by an unsaturated seven-carbon chain consisting of an α,β-unsaturated β-diketone. The presence of this specific group ensures the pH-dependent keto-enol tautomerism [53]. This keto-enol balance is very important for the physico-chemical and antioxidant properties of curcuminoids. Considering curcumin as an example, when it is in the enolic form both aromatic rings can interact, delocalizing the electrons present in the π orbitals. The chemical structure of curcuminoids also permits a wide range of beneficial and therapeutic effects as antimicrobial [54], anti-inflammatory [55,56], neuroprotective [57], and anticancer [58].

**Figure 2:**
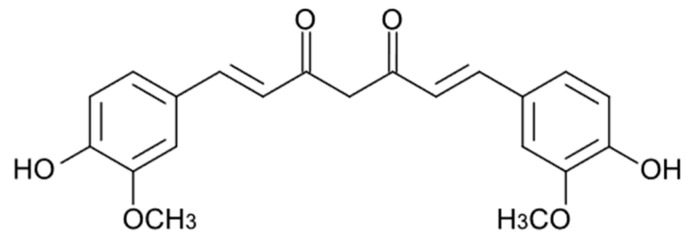
Chemical structure of curcumin.

Curcumin (CUR, Figure 2), the most studied of the curcuminoids, exerts its anti-inflammatory effects through upregulation of the peroxisome proliferator-activated receptor gamma (PPAR-γ) [59] and downregulation of NF-Kβ, thereby suppressing the subsequent synthesis of cytokines such as TNF-α, IL-1β, IL-6 and IL-8 and vascular endothelial growth factor (VEGF) [60]. It increases the plasma levels of superoxide dismutase (SOD) and glutathione peroxidase, enhancing the catalase activity and reducing the plasma levels of lipid peroxidase. CUR is able to participate in many signalling pathways by modulating several signalling molecules (e.g., pro-apoptotic proteins, COX-2, C-reactive protein, prostaglandin E2) and adhesion molecules [61].

The liphophilic-like structure confers to CUR a very low bioavailability after oral intake. Indeed, in healthy human subjects, after oral ingestion of 10 g, CUR showed an area under the curve (AUC) of 35.33 ± 3.78 μg/mL × hr and a Cmax of 2.30 ± 0.26 μg/mL, with a half-life (T_1/2_) of 6.77 ± 0.83 hr. In all subjects, CUR was detected in plasma primarily as glucuronide and sulfate conjugates, rarely as free CUR. Notably, no subjects reported mild or severe side effects [62].

### 4.1. In Vivo Animal Studies

The ability of curcuminoids to accelerate the resolution of inflammation and the reepithelization of the buccal mucosa lesions is enlightened in a prospective randomized controlled–blinded in vivo study performed by Schmidt et al. [63] in 2019. This study included 72 male golden Syrian hamsters affected by 5-FU induced OM and evaluated the effects of a topical treatment with (i) a mucoadhesive formulation containing curcuminoids (indicated as MCF and consisting of mucoadhesive gel containing CUR 20 mg/mL), (ii) a *Chamomilla recutita* L. fluid extract (indicated as Ad-Muc^®^), used as a phytotherapeutic positive control, and (iii) a placebo, corresponding to the CUR-free mucoadhesive formulation, each administered twice daily (0.5 g/dose) for 2 weeks. The treated groups (including the placebo) were compared with a further control group, which received 5-FU alone to induce OM and was then treated twice daily under identical conditions, but no substances were applied. The collected histopathological and macroscopic analyses showed that both MCF and Ad-Muc^®^ enhanced the tissue healing processes through the formation of new epithelial tissue thus covering the entire thickness of wounds. Moreover, the reepithelization process occurred rapidly as a result of the therapy. The so-treated hamster also exhibited lower angiogenesis, vascularization, and TGF-β1-labelling than the control group (animal treated with 5-FU alone) and the placebo group.

In 2022, Dvoretskiy et al. [64] evaluated the efficacy of polyphenols and nutrient topical administration in reducing radiotherapy-induced OM in Syrian golden hamsters. Eighty 5–6-weeks-old animals were subjected to a single dose of radiation (40 Gy) on day 0, resulting in OM after 6 days and achieving a peak (moderate or severe OM, WHO score ≥3) at days 14–16. Animals were evaluated up to 28 days and treated topically from day 1 to day 20, as follows: (i) receiving 0.25 mL of CUR 50 μg/mL in a 2% (*v*/*v*) ethanol in water solution; receiving 0.25 mL of CUR 100 μg/mL in a 2% (*v*/*v*) ethanol in water solution; (iii) control 1 receiving 0.25 mL of a 2% (*v*/*v*) ethanol in water solution; (iv) receiving 0.25 mL of Quercetin (QRC) 50 μg/mL in a 2% (*v*/*v*) DMSO in water solution; (v) receiving 0.25 mL of QRC 100 μg/mL in a 2% (*v*/*v*) DMSO in water solution; vi) control 2 receiving 0.2 mL of a 2% (*v*/*v*) DMSO in water solution; (vii) control 3 receiving 0.25 mL of water; (viii) receiving 0.2 mL of alanyl-glutamine dipeptide 30 mg/mL in water; (ix) receiving 0.2 mL of Arg/Gln/HMB 50 mg/mL in water; and (x) receiving 0.2 mL of Arg/Gln/HMB 100 mg/mL in water. With regard to CUR effectiveness, it is first remarkable that the animals in the control ethanol group experienced severe OM (score ≥3) for 35.5% of the experimental days, whereas this value decreased to 17.6% in the CUR 50 group. The OM score in the CUR 50 group was statistically reduced compared to the control from day 10, while the CUR 100 treatment showed significant results only after 28 days (QRC results are reported below in Section 5.5). According to the Mann–Whitney test, only CUR 50 was effective as it achieved almost 2 days of significant reduction during the period of study. In contrast, the amino acids-based treatments did not display the expected benefit probably due to low dosage.

### 4.2. Clinical Trials

The role of curcumin and curcuminoids in restoring buccal tissue in OM injuries induced by radio- and chemo-therapies is well established in the literature. Indeed, Normando et al. [65] published a systematic review in 2019 with the aim of evaluating the effects of turmeric and curcumin in the management of chemo- and radiotherapy-induced OM. In addition, the literature already collected by these colleagues will not be reported here again, even if several papers should meet the inclusion criteria here selected. Consequently, only the newest papers are reported below. It is relevant to emphasize that even if the considered time span here is limited (the last 4 years), several papers reporting on clinical trials continue to prove the usefulness of curcuminoids to prevent and treat chemo- and radiotherapy-induced OM. In 2020, Shah et al. [66] conducted a triple-blind clinical trial to assess the effectiveness and safety of a mouthwash containing CUR 0.1% (*w*/*v*), compared to a commercial one containing benzydamine 0.15% (*w*/*v*) (COOLORA™) for the treatment of radiotherapy-induced OM. Seventy-four patients with conclusive OSCC and undergoing radiotherapy (60–70 Gy) were divided into two groups receiving each 10 mL of CUR-containing mouthwash or the commercial formulation (control group) three times daily for 6 weeks. Patients were evaluated once a week and assigned an OM score (ranging from 0 to 4). Both the preventive and the curative effects of the treatment were assessed by evaluating the OM score. In particular, a score ≥1 was defined as the onset of OM, while a score ≤ 2 was defined as tolerable mucositis and ≥ 3 as intolerable mucositis. There were no significant differences between the two groups in terms of healing effect as both formulations were effective in the treatment of OM. However, CUR emerged as a more effective preventive strategy, as the use of the CUR-containing mouthwash reduced the risk of OM onset in almost 50% of patients and also showed a promising delay in the OM onset of 2 weeks. Furthermore, at the end of this clinical trial, there were no subjects in the CUR group characterized by an OM score ≥ 3.

In 2022, de Cássia Dias Viana Andrade et al. [67] investigated the role of both photobiomodulation and antimicrobial photodynamic therapy mediated by CUR and blue LED as an adjuvant strategy to manage OM in patients undergoing chemo- or radiotherapy. Thirty patients, with stable oral mucosa lesions and treated by radio- or chemotherapy, were randomly divided into three groups: (i) the control group receiving nystatin as standard treatment protocol; (ii) the group treated with low-level laser (λ = 660 nm, power: 100 mW) 3 times a week for 1 month; and (iii) the group treated with 450 nm blue LED and CUR (7.5 mg in 10 mL, prepared at the time of use) as photosensitizer once a week, for 1 month each. Patients were evaluated weekly for OM score and Candida albicans infection. Results showed that the participants who received the photo-treatments both in the presence and in the absence of CUR, showed a long-term (after 21 or 30 days of therapy) reduction of C. albicans infection, pain, and OM score (from the 21st day of treatment) when compared to the control group (which worsened after the 14th day and for the entire duration of the experiment). In particular, the CUR-based option was more effective than the photo-treatment alone in both minimizing infections and reducing OM symptoms. A double-blind clinical trial comparing the effect of three formulations loaded with mucosamin, chlorhexidine, and CUR in the treatment of OM was performed by Fardad et al. [68] in 2023. Specifically, 82 patients undergoing chemotherapy were divided into three groups treated with (i) commercially available Mucosamin^®^ oral spray (Professional Dietetics^®^, Milan, Italy) four puffs/day for two weeks; (ii) commercially available 0.2% (*w*/*v*) chlorhexidine mouthwash (Behsa^®^, Tehran, Iran) in 1:1 dilution for 1 min four times/day for two weeks; and (iii) 0.5% (*w*/*v*) CUR-loaded gel, four times/day for two weeks. Patients were assessed daily using the WHO score (0–4 scale) and OMAS scores (0–2 scale for erythema intensity; 0–3 scale for ulceration). Results showed that the severity of OM, as measured by WHO score, was significantly reduced in patients subjected to the CUR-based treatment compared to the other groups, with a complete restoration of the lesions after 4 days. On the other end, the other two approaches did not lead to complete recovery in the time period considered. CUR was also superior in terms of the OMAS score: the erythema disappeared after 4 days, compared to the 6 days required for the mucosamin-based treatment, while chlorhexidine did not resolve this symptom. Moreover, the ulcerative section parameter confirmed the complete tissue restoration after 5 days of treatment in the CUR group (tissue restoration was almost complete after 4 days), whereas mucosamin required 11 days of therapy and chlorhexidine was not fully effective again. In conclusion, all the formulations were found to be useful in the management the OM symptoms, but the use of CUR resulted in extremely rapid effects.

Another recent example of the efficacy of turmeric in the treatment of OM is the study published by Soni et al. [69] in 2022. In this paper, a double-blind clinical trial was performed on sixty patients undergoing chemotherapy. Patients were divided into three groups: (i) low dose of bioenhanced turmeric formulation containing curcuminoids and essential oil of turmeric (1 g/die, per os); (ii) high dose of bioenhanced turmeric formulation (1.5 g/die, per os); and (iii) placebo, for a total of 6 weeks with concurrent chemotherapy. OM severity, dysphagia, pain, dermatitis, and weight loss were the considered parameters evaluated during the study. Results highlighted that OM severity (grade 3) and OM-associated symptoms were significantly lower in both the two turmeric-treated groups. Additionally, the 1.5 g/die dose therapy was slightly more effective than the 1 g/die therapy, thus suggesting a real dose dependence. Furthermore, the groups treated with curcuminoids were more compliant, lost less weight, and did not require hospitalization compared to the placebo group. Due to the unfavourable physico-chemical properties of curcuminoids, their use in human studies could be limited in terms of the choice of formulation to be administered. As a result, the last 3 clinical studies reported below will all employ a nanostructured CUR formulation. The latter is SinaCurcumin^®^, a registered nanomicellar formulation designed for oral use containing curcumin, bisdemethoxycurcumin, and desmethoxycurcumin. This product was developed by the Nanotechnology Research Center of Mashhad University of Medical Science and marketed by Exir Nano Sina Company in Tehran, Iran. Each softgel capsule contains 80 mg of curcumin as nanomicelles having a CUR loading efficacy of almost 100% and significantly higher bioavailability after oral intake than the simple curcumin powder form [70,71]. A double-blind clinical trial was conducted by Delavarian et al. [72] in 2019 to assess the effects of SinaCurcumin^®^ on radiotherapy-induced OM. Thirty-two patients undergoing radiotherapy (50 Gy) were equally divided into study and control groups receiving SinaCurcumin^®^ capsules and lactose-loaded placebo tablets, respectively, once a day for 6 weeks. As observed, the study group displayed a one-week delay in the OM onset compared to the control group. However, once OM manifested, its severity gradually increased over time in both groups throughout the radiotherapy treatment in a time-dependent manner. Nevertheless, the beneficial effects of SinaCurcumin^®^ were undoubtedly confirmed as the study group showed less weight loss, probably related to the ameliorative effect of CUR leading to better food intake, and no grade 4 OM was registered. In contrast, almost 50% of patients in the control group manifested the highest WHO grade of mucositis.

Kia et al. [73] also evaluated the effectiveness of SinaCurcumin^®^ in 2021. Fifty patients undergoing chemotherapy (30–50 mg Cisplatin or 640–750 mg 5-FU) and/or radiotherapy (60–70 Gy) to treat head and neck cancers were selected and divided into two groups: the treated group received SinaCurcumin^®^ and the control one received placebo capsules, both twice daily for 7 weeks. The severity of mucositis and pain scores were evaluated after 1, 4, and 7 weeks of treatment. It was shown that OM was significantly more severe in the control group than in the treated group for the entire duration of the trial, while SinaCurcumin^®^ reduced the pain only at the end of the experiment. Furthermore, as expected, patients who received both chemo- and radiotherapy experienced a worsening of the OM clinical signs compared to the patients who underwent chemotherapy alone. CUR-based treatment was then confirmed to be effective in decreasing the severity and progression of OM, while also ameliorating patients’ quality of life, especially for those subjected exclusively to chemotherapy. The efficacy of SinaCurcumin^®^ was further demonstrated by Ramezani et al. [74] in 2023 through a randomized clinical trial comparing the latter oral dosage form with a CUR-loaded mouthwash. Forty-five patients affected by radiotherapy-induced OM (severity 1–3 in the WHO scale) and still ongoing radiotherapy were divided into three groups receiving (i) 40 mg/die of SinaCurcumin^®^, (ii) CUR mouthwash 0.1% (*w*/*v*), or (iii) placebo mouthwash, both liquid dosage forms for 1 min, three times daily. After 21 days, all the patients treated with CUR both topically and orally showed a reduction in OM severity, signs, and symptoms compared to the placebo group. In addition, between 15 and 33% of the subjects in the CUR-treated groups showed a complete resolution of the ulcer, while patients in the placebo group still had OM at the end of the trial. However, no statistical differences in the efficacy of the two CUR-loaded formulations were observed, probably due to the limited number of participants involved in the study as a result of the COVID-19 pandemic.

The studies collected and reported here (summarized in Table 1) clearly demonstrate the potential of curcuminoids in the prevention and treatment of chemo- and radiotherapy-induced OM, even more so when considering the limited time period covered here (the last 4 years). To further prove the consistency of the data in the literature, it is relevant to cite several recent meta-analysis reviews (e.g., Yu et al. 2020, Zhang et al. 2020, Dharman et al. 2021 [75,76,77]), which evaluated the results of numerous clinical trials, underlining the statistical significance of the enormous number of results reported over the years by several researchers.

Nevertheless, a further key point needs to be considered. The number of studies suggesting and proving that CUR and curcuminoids are an ideal strategy for the prevention and treatment of chemo- and radiotherapy-induced OM is certainly enormous. However, their actual clinical use is severely limited by some crucial problems related to low water solubility and bioavailability as well as relevant physico-chemical instability (due to light, alkaline pH, and high temperature) [78]. Indeed, in vivo protocols evaluating CUR- always required formulations (e.g., mouthwash, gel, capsule) to administer CUR, leading to the evaluation of its biological effects. The choice of both the vehicle and the physical state of the formulation should be well considered as they are of great importance. Indeed, they will significantly affect the therapeutic outcomes. For these reasons, various efforts should be made by drug delivery technologists to design novel, effective, and safe CUR-loaded drug delivery systems. Despite the oral administration of CUR being proven to be useful for the treatment of OM (see SinaCurcumin^®^ results), the beneficial effects of curcuminoids could be underestimated due to their low bioavailability. Thus, the design of innovative drug delivery systems for topical administration at the lesion site could be a smart strategy to improve stability, water solubility, active-tissue interaction, and thereby bioavailability, leading to increased efficacy at reduced doses while also avoiding any systemic side effects.

## 5. Flavonoids

Flavonoids are a large and important class of polyphenols, which, according to their chemical structure, can be further divided into flavones, flavonols, flavanones, flavonols, flavanols, anthocyanins, isoflavones, and chalcones. Flavonoids can be found in several fruits and vegetables and, recently, have been extensively studied due to their interesting bioactive properties. In nature, they can exist as aglycone and glycosylated forms. In general, dietary flavonoids, with the exception of flavanols, are found in glycosylated forms. Certainly, glycosylation influences physico-chemical properties such as water solubility, while the literature confirms that it does not affect biological activity [40]. Furthermore, flavonoids can also be found as oligomers or esterified with gallic acid [79].

**Figure 3:**
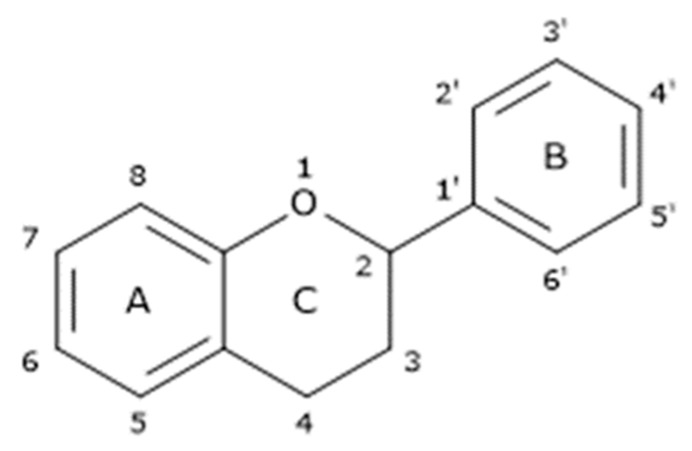
Flavonoids’ general chemical structure.

Flavonoids are chemically characterized by a core structure constituted by a 15-carbon skeleton (Figure 3) consisting of two fused rings (benzene -A- and pyran -C) linked with a catechol or phenyl group (B) [80].

This conjugated polyunsaturated structure is responsible for their antioxidant activity due to the delocalization and stabilization of free radicals, which depends on various factors, e.g., the position of the catechol/phenyl ring with respect to the pyran one and number of hydroxyl groups on catechol rings. These structural factors also influence bioavailability and biological activity [80]. Currently, flavonoids are well known for their multiple health-promoting effects: anti-inflammatory, immunomodulatory, antibacterial, antiviral, anti-aging, cardio and neuroprotective, anticancer, and antidiabetic [40,81,82]. All these biological activities make them interesting for nutraceutical, cosmetic, and pharmaceutical applications. In particular, they have recently been proposed as adjuvants for the treatment of some oral diseases caused by high inflammation and oxidative stress (e.g., enhanced ROS production) such as chemo- and radiotherapy-induced OM.

The literature reports both on the efficacy of isolated flavonoids (e.g., hesperidin, epicatechin, epigallocatechin-3-gallate, apigenin, and Quercetin) and complex mixtures of plant-derived flavonoids. Therefore, this section will be further subdivided to present the studies of each single molecule as well as those of the complex matrices.

### 5.1. Hesperidin

**Figure 4:**
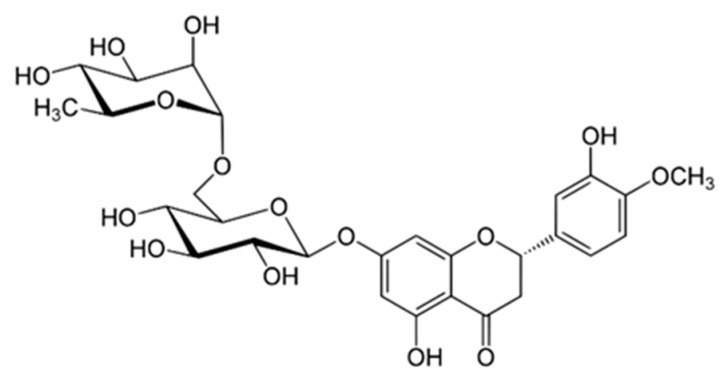
Chemical structure of Hesperidin (HSP; (2S)-3′,5-Dihydroxy-4′-methoxy-7-[α-L-rhamnopyranosyl-(1→6)-β-D-glucopyranosyloxy]flavan-4-one).

Hesperidin (HSP, Figure 4, (2S)-3′,5-Dihydroxy-4′-methoxy-7-[α-L-rhamnopyranosyl-(1→6)-β-D-glucopyranosyloxy]flavan-4-one) is a β-7-rutinoside belonging to the subclass of flavanones. It is particularly abundant in the peel of Citrus fruits (e.g., lemon and grapefruit) [83,84] and is derived from the reaction between Hesperetin (position 7) and the disaccharide rutinose (6-O-α-L-rhamnosyl-D-glucose) via O-glycosidic bond.

HSP has a wide range of biological activities such as antihyperlipidemic [85], antidiabetic [86], cardioprotective [87], antioxidant, and anti-inflammatory properties [88], making this compound a valid agent for the prevention and treatment of OM. Nevertheless, despite the presence of a disaccharide, HSP suffers from low water solubility leading to low bioavailability, as well as degradation by gut microbiota, both of which dramatically limit its clinical use [87]. HSP bioavailability is estimated to be about 20% as it possesses poor permeability from the gastrointestinal tract together with being the substrate of P-glycoprotein [89].

As reported by Manach and colleagues who compared the data from 97 bioavailability studies of the main natural polyphenols, the HSP pharmacokinetics parameters resulted within the following ranges: AUC 1.9–4.1 µmol h/L, Tmax 5.4–5.8 h, and Cmax 0.21–0.87 µmol/L after an oral dose of 50 mg [90]. Issues related to low water solubility are also quite relevant for in vivo/clinical evaluations. An innovative approach to overcome this problem is represented by the biosynthetic transglucosylation, performed through bacteria [91]. This process is useful to obtain a molecule that retains the same biological properties of HSP while displaying enhanced solubility in aqueous media, and thus better bioavailability. An example from the literature is the in vivo studies reported by Yoshino et al. [92] in 2016. They employed α-glucosyl hesperidin (α-G-HSP) and evaluated its preventive effect against 5-FU-induced OM. This study was based on the hypothesis that the OM symptoms are exacerbated by the massive inflammatory response caused by 5-FU treatment. Four-week-old male Syrian golden hamsters were divided into 3 different groups: group 1 received no treatment (control), group 2 was treated with a placebo, and group 3 was treated with α-G-HSP (1 mg/mL ad libitum) from 5 days before 5-FU intraperitoneal injection (60 mg/kg) leading to OM, until 16 days. Results showed that pre-treatment with α-G-HSP significantly reduced OM signs. In particular, smaller ulceration areas were observed when compared to the control and placebo groups. This effect was explained by a significant inhibition of ROS species which are involved in the initiation of lipid peroxidation leading to OM. As a consequence, α-G-HSP could be a valid agent for the prevention of chemotherapy-induced OM. However, further studies are still required to confirm the same potential in humans.

### 5.2. Epicatechin

**Figure 5:**
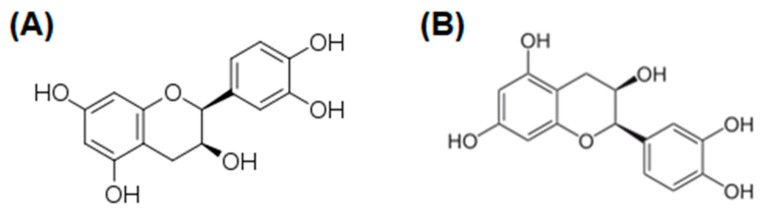
Chemical structures of (**A**) (+)-Epicatechin ((2S,3S)-2-(3,4-Dihydroxyphenyl)-3,4-dihydro-2H-chromene-3,5,7-triol) and (**B**) (−)-Epicatechin ((2R,3R)-2-(3,4-Dihydroxyphenyl)-3,4-dihydro-2H-chromene-3,5,7-triol).

Catechins are the main polyphenols found in *Theobroma cacao* L., *Arachis hypogea* L., and green tea (*Camellia sinensis* L.). They belong to the subfamily of flavanols and exhibit direct antioxidant activity due to lipid peroxidation inhibition, free radical scavenging activity, and modulation of ROS production pathways [93,94]. Furthermore, they also exert antimicrobial [95,96] and anti-inflammatory activities [96]. Catechins are characterized by a peculiar chemical structure that differs from many other flavonoids because of the lack of unsaturation in the pyranose ring and the substitution of the keto group with a hydroxyl in position 3. For this reason, they are also called flavan-3-ols. The presence of two chiral carbons (positions 2 and 3) leads to the existence of diastereomers. In particular, two of them are called Epicatechin and exist as (+)-Epicatechin (Figure 5A) with a (2S,3S)- configuration and its enantiomer (−)-Epicatechin (Figure 5B) [94].

Like most other polyphenols, (−)-Epicatechin (EC) suffers from low bioavailability after in vivo administration, mainly due to its chemical instability in the gastroenteric environment and massive first-pass effect [97]. The pharmacokinetic parameters evaluated on male human volunteers receiving an oral single dose of 200 mg of EC resulted in AUC of 122.4 ± 9.0 µg/L × h, Cmax of 34.5 ± 5.3 µg/L, Tmax 1.0 ± 1.0–2.0 h, Ke of 0.277 ± 0.041 h^−1^ and T_1/2_ of 2.5 ± 0.4 h [98]. Consequently, several novel approaches aimed at enhancing EC bioavailability have been proposed, such as nanoencapsulation [99].

Shin Y.S. et al. [100] evaluated the effectiveness of EC on radiotherapy-induced OM both in vitro and in vivo. The preliminary in vitro studies, necessary prior to the development of the in vivo protocol, were performed on human keratinocytes and highlighted a significant dose-dependent cytoprotective effect against ROS generation, as well as inhibition of the radiation-induced expression of pro-apoptotic and pro-inflammatory factors such as p-JNK, p-38, and cleaved caspase-3. To in vivo evaluate EC effects, 32 Sprague-Dawley rats (females, 6 weeks old) were divided into four groups: the control group, two groups receiving either EC (2 mM solution; administered orally 3 times a day; 100 μL/dose) or radiation (30 Gy) and, finally, the group treated with a combination of both (radiation: 30 Gy; EC was orally administered 3 times daily, 100 μL/dose of a 2 mM solution) for a total of 23 days. As a result, the EC-based treatment inhibited radiation-induced apoptosis and minimized the damage to the epithelial cell layer. The generation of ROS and NOX-3 protein was significantly reduced in rats’ tongue and buccal mucosa by the EC treatment compared to rats receiving radiotherapy alone. Thus, it can be affirmed that the strong scavenging activity of EC might have a key role in preventing and delaying the onset of OM, reducing pain and ulcer size, thereby facilitating the healing process. Furthermore, radiotherapy resulted in 100% of the rats dying, while the combination of EC + radiation led to 50% of animals surviving at the end of the experiment. Clearly, further tests are still needed to confirm these promising early results.

### 5.3. Epigallocatechin-3-gallate

**Figure 6:**
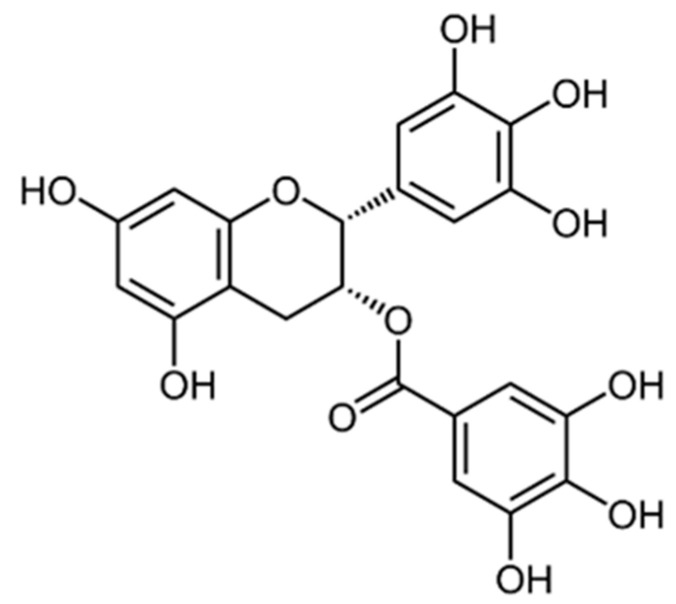
Chemical structure of Epigallocatechin gallate (EGCG; (2R,3R)-3′,4′,5,5′,7-Pentahydroxyflavan-3-yl 3,4,5-trihydroxybenzoate).

Epigallocatechin-3-gallate (EGCG, Figure 6, (2R,3R)-3′,4′,5,5′,7-Pentahydroxyflavan-3-yl 3,4,5-trihydroxybenzoate) is the most abundant flavonoid found in *C. sinensis* L., and it is responsible for several of the health benefits associated with Green tea consumption [101]. It belongs to the class of flavanols and, chemically, it is an ester between epigallocatechin and gallic acid. It has excellent scavenging activity against ROS species [102], and possesses a wide range of biological effects such as anti-inflammatory [103], wound healing [104], and neuroprotective [105]. EGCG also exerts chemopreventive activity by reducing cancer cell invasion and migration through different mechanisms such as reduction of matrix metalloproteinases, expression of urokinase-type plasminogen activator, and stimulation of apoptosis and autophagy [106].

Despite its hydrophilic-like structure, EGCG suffers from low bioavailability after oral ingestion as it is preferentially excreted through the bile to the colon after oral absorption. Indeed, as reported, human volunteers receiving EGCG (2 mg/kg body weight) orally showed AUC of 213.7 ± 86.4 ng/mL × h, Cmax of 34.71 ± 22.87 ng/mL, and T_1/2_ of 3.70 ± 2.22 h [107]. In 2019, Zhu et al. [108] conducted a phase I clinical trial to assess the safety and efficacy of an EGCG-containing mouthwash in patients with head and neck cancer undergoing radiotherapy (66–72 Gy daily) alone or in combination with chemotherapy. Specifically, 20 patients were asked to gargle with 15 mL of a mouthwash containing increasing doses of EGCG (440 μM, 880 μM, 1320 μM, 17,602 μM, and 2200 μM) three times per day for 5 min for 8 weeks. All patients were followed during and after treatment. Results showed that the EGCG-loaded mouthwash was well tolerated and significantly reduced the pain and the OM score, although no significant differences were observed by varying the employed ECGC dose, probably due to the limited number of involved subjects. After treatment, more than 50% of the patients reported an improvement in symptoms and experienced less pain than before EGCG application. Moreover, no patients showed a high OM score (>3) or required feeding tube placement/liquid diet after EGCG application. The authors acknowledged that this study may be controversial as it involves a small cohort of patients, obviously leading to some limitations. Nevertheless, this trial highlights the potential benefits of the use of EGCG to promote both lesion healing and patients’ quality of life and suggests a further phase II clinical trial using a recommended dose equal to 1760 μM. The latter is significantly higher than the dose currently used in an oral or topical formulation, but is considered necessary to achieve therapeutic success as the salivary secretion is greatly increased in response to the taste stimuli provided by the EGCG mouthwash, thus diluting the active ingredient. Considering the potentiality of EGCG, the latter could be a useful molecule to be embedded into a suitable ad hoc designed buccal drug delivery system in order to minimize the required dose while increasing efficacy.

### 5.4. Apigenin

**Figure 7:**
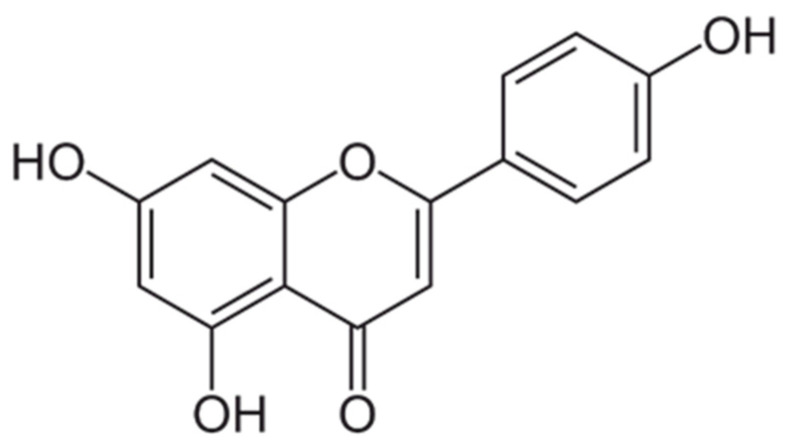
Chemical structure of Apigenin (APG; 4′,5,7-Trihydroxyflavone).

Apigenin (APG, Figure 7; 4′,5,7-Trihydroxyflavone) is the main characteristic flavone present in *Achillea millefolium* L. and *Chamomilla recutita* L. Important food sources of APG are also dietary plants such as parsley (*Petroselinum crispum*), celery (*Apium graveloens*), and artichoke (*Cynara cardunculus*). Knowledge regarding the bioavailability of APG in humans is limited due to its low solubility and difficulties in the detection of APG metabolites in plasma and urine. A recent study from 2022 in which 191 μmol APG in capsules as a dietary supplement were administered to volunteers, highlighted that neither APG nor its metabolites were detected in plasma collected until 6 h after the ingestion, whereas a total of 0.9 μmol of metabolites, equal to 0.5% of the ingested dose, were present in urine excreted within 24 h of flavone intake [109].

Despite APG was per se poorly absorbed by the gastrointestinal tract, the aforementioned APG-rich plants have been widely used for centuries as traditional medicines due to their spasmolytic [110], anti-inflammatory [111], and antimicrobic properties [112]. Considering these interesting activities, a possible application of APG as a therapeutic agent for the management of OM should be considered.

In 2016, Molina Prats et al. [113] conducted a double-blind controlled pilot study comparing the effects of gavage administration of APG or Dexamethasone for the treatment of 5-FU-induced OM. The study was performed on male Syrian golden hamsters and evaluated any changes affecting the oral mucosa. Thirty-six animals were randomly divided into three groups: (i) the control group was treated with acetic acid (1 mg/Kg), (ii) the second group received both acetic acid (1 mg/Kg) and APG potassium salt solution (K-APG; 40 mg/Kg), as it possesses higher water solubility than APG [114], and (iii) the third group was treated with acetic acid (1 mg/Kg) and dexamethasone water solution (1 mg/Kg). The experiment was carried out for a total of 14 days and OM was induced in all the groups through intraperitoneal injection of 5-FU (60 mg/kg) at days 0 and 2. Histomorphometric and immunohistochemical analyses were performed on days 5, 7, 10, and 14 and showed that the initial evolution of OM was similar for the 3 groups, while substantial differences in terms of OM severity, lesion size, and wound-healing process were observed throughout the entire experiment. Specifically, the control group experienced a rapid onset of OM together with high microbial infection, which worsened up to day 10. In contrast, both the treated groups showed a significant reduction in OM scores up to day 10, with the K-APG group showing the lowest scores. After day 10, no statistically significant differences were highlighted due to the normal healing process also occurring in the control group. However, it isn’t doubtful that K-APG reduced the inflammation infiltrate and edema and accelerated tissue restoration at a much earlier stage, thus proving its potential usefulness in the prevention and treatment of chemotherapy-induced OM.

Nevertheless, APG is susceptible to degradation and high metabolism which limit its clinical use. For these reasons, there is a clear need for new technological approaches to improve solubility, bioavailability, and stability.

### 5.5. Quercetin

**Figure 8:**
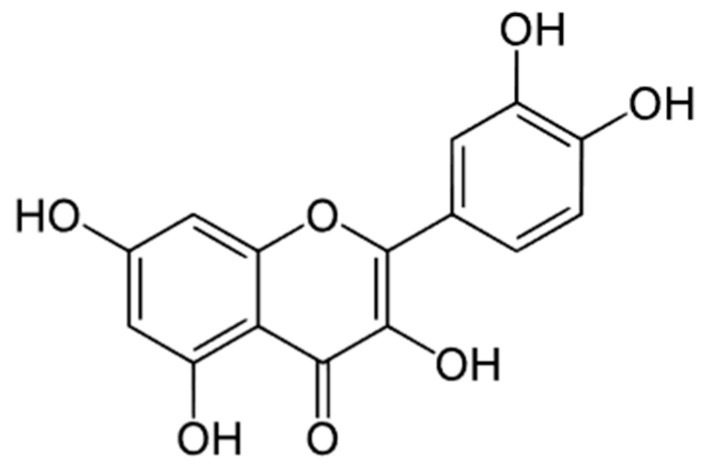
Chemical structure of Quercetin (QRC; 3,3′,4′,5,7-pentahydroxyflavone).

Quercetin (QRC, Figure 8; 3,3′,4′,5,7-pentahydroxyflavone) is a phytoestrogen-like flavonoid, belonging to the subclass of flavonols. It can also be found in different glycosylated forms, such as Rutin (quercetin-3-O-rutinoside).

It is widely distributed in several foods and plants such as *A*. *millefolium* L., *Calendula officinalis* L., *C. recutita* L., *C. sinensis* L., and *Vitis vinifera* L. The chemical structure of QRC is characterized by four hydroxyl groups (3 -OH groups and one related to the keto-enolic tautomerism) on the benzo-dihydropyran-fused rings that are responsible for its biological activities [115]. According to Mbikay et al. [116], QRC acts as an antioxidant agent both directly as a scavenger for ROS species as well as indirectly by inhibiting LDL oxidative damage and thus the lipid peroxidation process. QRC also manifests anti-inflammatory and immunosuppressive effects [117], anticancer [118], and wound-healing properties [119], and it can promote cell proliferation and osteogenic differentiation [120]. This wide and polyhedric spectrum of biological activities makes QRC a promising therapeutic agent also useful in the odonthostomatology field and particularly in the management of OM.

QRC is characterized by low water solubility and high pH-related instability, which compromise its bioavailability after oral intake. Furthermore, its gastrointestinal absorption is limited by both the high rate of efflux and the formation of conjugates in the enterocytes. Moon et al. [121] estimated in healthy human volunteers that after ingestion of 500 mg three times daily, QRC was detected in plasma mainly as methyl, glucuronide, and sulfate conjugates and in very low amounts as unchanged aglycone. The pharmacokinetic parameters for QRC aglycone ranged in AUC 2.25–182 ng/mL × h, Cmax 0.49–39.9 ng/mL, Tmax 1–5 h, T_1/2_ 0.956–12.5 h whereas for metabolites they ranged in AUC 990–6960 ng/mL × h, Cmax 161–1210 ng/mL, Tmax 1–6 h. Dvoretskiy et al. [64] evaluated in 2022 the efficacy of the daily topical administration of polyphenols and nutrients such as Curcumin (see above, Section 4), Quercetin, and amino acids in 80 male Syrian golden hamsters (5–6 weeks old) affected by radiotherapy-induced OM. Specifically, two different QRC doses were tested (50 and 100 μg/mL) in a 2% (*v*/*v*) DMSO in water solution which was administered topically (0.25 mL) and compared to a control receiving 0.25 mL of a 2% (*v*/*v*) DMSO in water solution. Regarding QRC effectiveness, it is relevant to report that the animals in the control DMSO group experienced severe OM (score ≥ 3) for 41.3% of the experimental days, whereas this value decreased to 27.6% and 25.0% in the QRC 50 and QRC 100 groups, respectively. However, only QRC 100 was considered effective, according to the Mann–Whitney test.

The protective role of QRC against mucosal injury due to radiotherapy was also investigated by Zhang J. et al. [122] in 2021. They tested 25 female C57BL/6 mice (6–8 weeks old) as follows: (i) the control group (no IR) did not receive any treatment, (ii) the single dose irradiation group (S-IR) received one shot 25 Gy radiotherapy, (iii) the fractioned dose irradiation group (F-IR) was subjected to 8 Gy/day radiotherapy for 3 days, (iv) the S-IR-QRC group received a one-shot 25 Gy radiotherapy together with QRC, and (v) the F-IR-QRC group received 8 Gy/day radiotherapy for 3 days along with QRC. Animals in the QRC groups were pre-treated by QRC oral administration (300 mg/kg/die) from one week prior to the irradiation and until 8 days post radiotherapy. Mice in both the QRC groups showed improved clinical conditions manifested by less body weight loss, reduced ulceration areas (which resulted in one order of magnitude smaller than those of the S-IR and F-IR groups), and minimal oral mucosa epithelial damage. Specifically, the epithelium was only slightly disordered and thinner after QRC treatment compared to the control group, while radiotherapy alone induced mucosal integrity loss and strong infiltration of inflammatory cells. Furthermore, the histochemical analyses showed a dramatic reduction of wellness proliferative markers normally present in the control groups (e.g., Ki-67 and PCNA) after irradiation. However, no evident reduction of these markers was observed in the QRC groups. Additionally, radiotherapy enhanced the expression of two markers related to cell senescence (P-21 and P-16) which were also inhibited by QRC, suggesting its role in both stimulating cell growth and inhibiting cell senescence and death. Finally, the authors observed that QRC significantly inhibited ROS production as well as the expression of the main pro-inflammatory cytokines (e.g., IL-6, IL-1β, TNF-α, TGF-β1). Thus, this study confirms QRC as a valuable preventive agent for managing radiotherapy-induced OM.

In 2021, Lotfi et al. [123] evaluated the preventive and therapeutic effects of QRC against chemotherapy-induced OM when administered free or in the form of a nanoemulsion. Thirty-six adult Albino male mice were randomly divided into 6 groups: (i) untreated control group; (ii) mucositis group, presenting OM due to 5-FU administration (300 mg/kg at the 6th day of experiment); (iii) pre-treatment nano-QRC group receiving QRC-loaded nanoemulsion (corresponding to a QRC dose equal to 5 mg/kg/die) from the 2nd to the 6th day of experiment; (iv) pre-treatment QRC group receiving QRC (5 mg/kg/die) from the 2nd to the 6th day of experiment; (v) post-treatment nano-QRC group receiving QRC-loaded nanoemulsion (corresponding to a QRC dose equal to 5 mg/kg/die) from the 7th to the 13th day of experiment; and (iv) post-treatment QRC group receiving QRC (5 mg/kg/die) from the 7th to the 13th day of experiment. Considering the general condition of the animals, QRC-based treatments reduced the weight loss observed in the mucositis group, and, approximately 7 days after mucositis induction, the weight started increasing. At a macroscopic level, the mucositis group was characterized by reduced tongue mucosa integrity, haemorrhage, inflammatory cell infiltration, and hyalinization, whereas these signs were not observed in the groups receiving QRC. The histochemical analysis carried out on blood samples highlighted that the pre-treatment with QRC significantly reduced the blood amount of Malonaldehyde (MDA), whereas both QRC-based pre- and post-treatments increased levels of the superoxide dismutase (SOD) and catalase (CAT). Results suggested that QRC could be useful for both prevention and treatment of OM. However, a deeper study is still necessary to avoid unexpected effects, as the authors suggest that any difference between the QRC-free and the nano-QRC is related to different cell permeability which could lead to the necessity of employing lower nano-form doses to achieve the desired effect without risks.

QRC has been also evaluated in humans by Kooshyar et al. [124] in 2017. This research team performed a randomized placebo-controlled double-blind clinical trial involving 23 adult patients undergoing high-dose chemotherapy (predominately Cytarabine or Daunorubicin, but also Vincristine, Cyclophosphamide, Plasil, Idarubicin, Fludarabine, Adriamycin, and L-asparaginase). Patients were divided into control and treatment groups and treated with a capsule containing 250 mg of lactose as a placebo or the same amount of QRC hydrate, respectively, twice a day from the onset of chemotherapy for a total of 4 weeks. The results showed that the incidence of mucositis was lower in the treated group than in the placebo one (30% vs. 60%, respectively). Despite the limitations of this clinical trial (small cohort of patients involved and poor oral health conditions limiting the dose of QRC to be evaluated), these results already open the possibility of considering QRC as a valid strategy to prevent and treat chemotherapy-induced OM. Hence, future studies are still required to assess the most effective QRC dose for this purpose in humans.

### 5.6. Flavonoids-Rich Plants and Their Use in OM

As previously reported, flavonoids are widely distributed in several plants and their derivatives. Among them, Aloe Vera [125], *C. recutita* [126,127], *A. millefolium* [128], Green tea [129], propolis, and honey [130] are the most known.

The natural protective and positive effects of polyphenols in the plant’s homeostasis are well known and have led several researchers over the years to consider their potential beneficial and therapeutic effects on human health. In this regard, the synergistic effect of the entire pool of bioactive molecules available from the plant kingdom (including flavonoids) should also be taken into account, as the overall beneficial effect could not simply be the sum of the activities of each individual component, but it could be enhanced by a complex and fine mixture, effectively designed by nature. In light of these considerations, numerous in vivo studies and some clinical trials have recently been carried out to evaluate the effectiveness of flavonoids-rich plant extracts in the prevention and treatment of chemo- and radiotherapy-induced OM.

#### 5.6.1. In Vivo Animal Studies

In 2017, Farrokhi et al. [131] evaluated the effects of topical gels containing a hydro-alcoholic extract of *Hypericum perforatum* and/or a *Pistacia atlantica* oil extract in promoting the wound healing process after OM induction. The well-known herbal plant *H. perforatum* is considered a rich source of polyphenols and its wide range of biological effects could be attributed to its multiple flavonoid compounds such as Rutin, Quercetin, Apigenin, Hypericin, Hyperforin, Chlorogenic acid, and Xanthone derivatives [132]. This rich pool of natural actives has been highlighted to possess antibacterial and anti-inflammatory activities, a protective role against oxidative stress and lipid peroxidation, as well as wound-healing properties due to stimulation of fibroblasts leading to promoted collagen production [133]. *P. atlantica* fruits have also long been employed due to their anti-inflammatory, wound-healing, expectorant, antidiabetic, and anticancer properties [134,135]. These are related to the high content of saturated and unsaturated fatty acids as well as triterpenoids, tocopherols, tocotrienols, and phenolic compounds (e.g., gallic acid). Sixty male golden hamsters (8–10 weeks old) were subjected to chemotherapy (5-FU, 60 mg/kg on days 0, 5, and 10; evaluation of OM severity on day 12) to induce OM and then the healing effect of both *P. atlantica* and *H. perforatum* extracts was evaluated. Animals were divided into 5 groups: groups 1, 2, and 3 were treated topically by covering the entire wound with a gel containing 10% (*w*/*v*) of hydro-alcoholic extract of *H. perforatum* or a gel containing 10% (*w*/*v*) of *P. atlantica* oil extract or a combination thereof, respectively; group 4 (placebo) received only the base gel and group 5 did not receive any treatment (control group). After 15 and 18 days of the experiment, the lesions were examined macroscopically, and excised biopsies were analysed for histopathological and biochemical assays. All the groups treated with the plant extracts, particularly group 3, exhibited lower microscopic and macroscopic scores compared to the other groups indicating that the healing process was promoted by the natural actives. Levels of two markers associated with oxidative stress such as myeloperoxidase and malondialdehyde were lower in the animals treated with the mixed gel than the other groups, suggesting that the plant’s extracts may inhibit lipid peroxidation and protect from oxidative stress. This study highlights that daily use of *H. perforatum* in combination with *P. atlantica* can accelerate normal healing, making it suitable to manage OM.

Also, the protective role of *C. officinalis* extract in the management of chemotherapy-induced OM was investigated by Tanideh et al. [136] in 2013. *C. officinalis* is a rich source of coumarins, carotenoids, amino acids, alkaloids, flavonoids, tannins, triterpenoids, and phenolic acids and has been used for centuries as a topical and oral remedy due to its bactericidal [137], anti-inflammatory [138], antioxidant [139], and wound-healing properties [140]. Sixty young male (6–8 weeks old) golden Syrian hamsters were subjected to intraperitoneal injections of 5-FU (60 mg/kg dose on days 0, 5, and 10, evaluation of OM severity on day 12 to induce OM and then randomly divided into 4 groups treated once a day from day 2 to day 17: groups 1 and 2 were treated topically with a sodium carboxymethyl cellulose-based mucoadhesive gels containing a hydroalcoholic extract of *C. officinalis* 5% or 10% (*w*/*v*), respectively, group 3 and 4 were the placebo (base gel) and the control group (no treatment), respectively. Histopathological analyses were executed on exerted cheek punches. Results showed a significant difference between treated groups and control/placebo groups. In particular, histopathologic signs including severe vascular ingurgitation, inflammatory infiltration, extensive ulceration, and haemorrhagic areas were decreased in both the 5% and 10% *C. officinalis* gel groups. Furthermore, the treatment with the two extract concentrations significantly reduced OM symptoms and accelerated the wound-healing process when compared with the other groups, in a dose-dependent manner.

**Figure 9:**
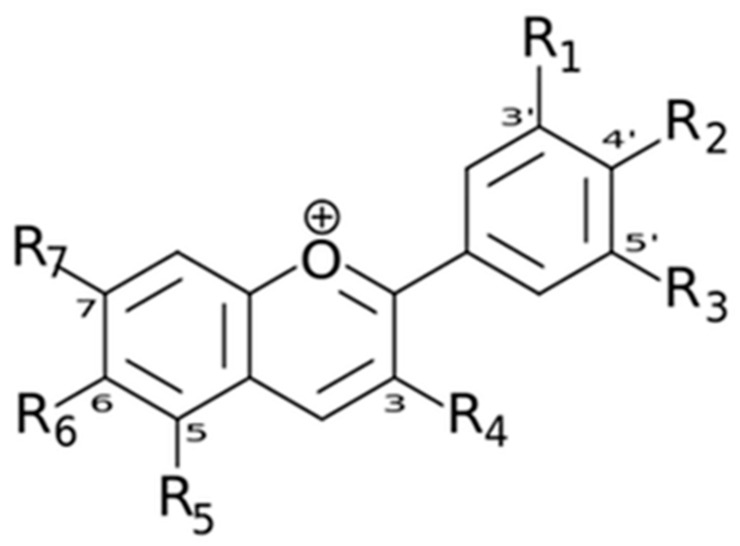
Anthocyanins (ANTs) general chemical structure.

Anthocyanins (ANTs, Figure 9) belong to flavonoids and are known as they determine the colours of fruits and flowers (from pink to blue) in several plants. They exhibit different therapeutic effects including cardioprotective, neuroprotective, and anti-inflammatory. Moreover, they are strong antioxidants, able to reduce the lipid peroxidation process and ROS generation, thus mitigating their negative effects on the oral mucosa environment [141].

ANTs are widely used as natural colorants for foods and are obtained from red vegetables and fruits such as black rice and grapes. Among ANTs-rich plants, *Vaccinium myrtillus* L. (bilberry) has been highlighted for its in vitro activity toward ROS generation and NF-kB activation. In 2013, Davarmanesh et al. [142] published an in vivo study aimed at evaluating firstly the effective dose and then the therapeutic potentiality against OM of a standardized bilberry extract containing 36% (*w*/*v*) of glycosylated ANTs as a preventive treatment against 5-FU induced OM in hamsters. The preliminary assessment involved 12 healthy adult golden Syrian hamsters which were randomly divided into 3 groups receiving ANTs standardized bilberry extract in deionized water by gavage once a day for 10 consecutive days at a final dose of 7.5 mg/mL (50 mg/kg), 45 mg/mL (300 mg/kg), and 90 mg/mL (600 mg/kg), respectively. On days 6 and 7, animals were subjected to intraperitoneal injections of 5-FU (40–60 mg/kg) to induce OM. The best outcomes were observed in the second group (300 mg/kg of standardized bilberry extract) and these data were employed for the further deeper randomized controlled trial. Subsequently, 24 further animals were randomly divided into other 3 groups: group 1 was the negative control as it did not receive pre-treatment or chemotherapy; group 2 was the positive control as it did not receive bilberry while subjected to 5-FU treatment; and, finally, group 3 was pre-treated with the bilberry extract-loaded solution (45 mg/mL, corresponding to 300 mg/kg of dose) by gavage once a day for 7 days, and, afterwards, subjected to chemotherapy (5-FU 60 mg/kg by intraperitoneal injections on days 4, 9, and 14). Both macroscopic (general health status and weight loss) and microscopic (histopathology) evaluations were conducted and recorded. The authors observed that the group pre-treated with the bilberry extract displayed lower weight loss and less severe clinical signs and changes in the oral mucosa than the positive control group. Furthermore, the pre-treatment with the bilberry extract led to a reduction of the OM severity, while the positive control group showed severe clinical manifestations, such as ulceration, abscess formation, and haemorrhage. These results prove that ANTs-rich extracts from plants such as V. myrtillus could be useful in preventing chemotherapy-induced damage affecting the oral mucosa.

**Figure 10:**
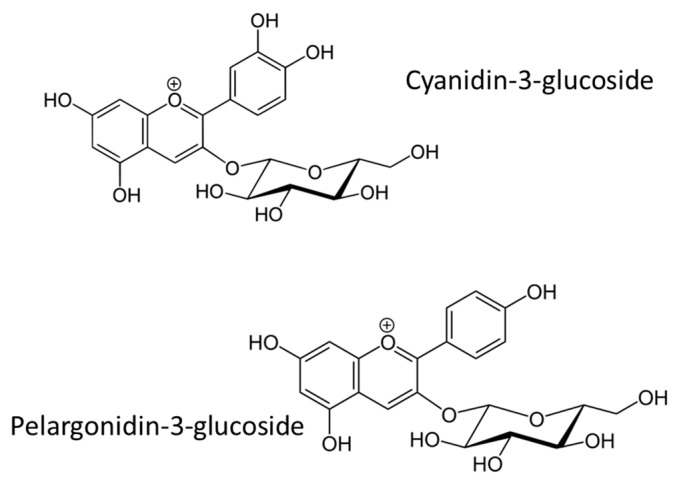
Main polyphenolic actives of *Oryza sativa* L.

The immunomodulatory effect of ANTs was observed by Tancharoen et al. [143] in 2018 by evaluating an ANTs-rich extract obtained by *Oryza sativa* L. (Figure 10) on a rat model.

Healthy male Wistar rats were divided into four groups: groups A and B were the negative and positive controls, respectively (untreated or subjected to intraperitoneal injections of 5-FU 60 mg/kg on days 7, 12, and 17), and groups C and D were gavage-fed once a day with 500 mg/kg or 1000 mg/kg of the *Oryza sativa* L.-obtained ANTs-rich extracts from day 1 to day 29, respectively, and received both the chemotherapy according to the other groups’ protocol. Animals were monitored daily, weighed every two days, and then subjected to histochemical analysis, and immunohistochemistry for p50 and p65 subunits of NF-κB and HMGB1 were conducted. All the animals in the positive control group showed macroscopically massive tissue granulation and visible ulcerated haemorrhagic areas. Additionally, nuclear localization of NF-κB and cytosolic overexpression of HMGB1 were detected in cells belonging to the stratum granulosum and spinosum of the oral epithelium. These markers were reduced in groups C and D suggesting the positive effect of the pre-treatment with ANTs, in a dose-dependent manner. In addition, in the late stage of the experiment, the ANTs-treated groups showed better tissue conditions in terms of vascular ingurgitation, ulceration, haemorrhage, and prevalence of re-epithelialization areas. These interesting wound-healing properties were also confirmed by the literature reporting strong anti-inflammatory activity of ANTs, making them ideal candidates for the treatment of OM [144]. All these results suggest that further human investigation and clinical trial protocols are required.

Among the well-known flavonoids-rich plants, green tea is undoubtedly one of the most studied over the centuries for its beneficial properties in human health. The main beneficial effects of this plant are related to its antioxidant and anti-inflammatory properties which are determined by the huge presence of flavonoids and particularly catechins (e.g., EGCG). Several works demonstrated the role of these molecules in (i) preventing lipid peroxidation [145]; (ii) antagonizing the oxidative stress and the production of nitric oxide (NO) radicals through the reduction of the AP-1 and NF-κB activities, inhibition of AKT signalling pathway and up-regulation of AMPK signalling pathway [146]; and (iii) controlling the Nrf2 genes. Moreover, they can also inhibit the overproduction of pro-inflammatory cytokines and mediators, including TNF and IL-6, and suppress c-Jun NH2-terminal kinase and p38 MAPK activities [145]. Consequently, the variegate pool of compounds present in green tea may act by regulating multiple mechanisms involved in the onset of OM.

In 2014, Al-Refai et al. [129] investigated the cytoprotective effect of green tea extract in order to prevent chemotherapy-induced OM. Firstly, a pilot study was performed to obtain a chemotherapy-induced OM model by administration of Methotrexate (MTX) in rats. Twelve Albino rats were randomly divided into different groups subjected to intraperitoneal injections of a single dose of MTX at increasing concentrations from 10 to 80 mg/kg. After 24 h, the animals were sacrificed, and the oral mucosa was investigated. Results showed that the highest MTX dose determined the maximum toxic effect and for this reason, this dose was chosen for the following in vivo experimental protocol. Further, 24 rats were randomly divided into four groups: (i) a positive control group, receiving intraperitoneal injection of MTX 80 mg/kg, (ii) a green tea group, treated by gavage twice a day with aqueous green tea extract (dose per day: 40 mg/kg) together with concomitant MTX treatment, (iii) green tea control group, receiving only the aqueous green tea extract (40 mg/kg/die), and (iv) negative control group, receiving only water. The experiment was carried out for 5 days and MTX was administered 72 h before the day count started. The green tea aqueous extract was obtained starting with a household method of tea making (5 g of dried green tea leaves in 60 mL of boiling water). The dispersion was filtered and dried until a paste was obtained and then dissolved in water to achieve the chosen final concentration. As the authors reported, MTX reduced the epithelium thickness, inducing the destruction of the basal lamina. In addition, a large number of subepithelial inflammatory cells and congested blood vessels appeared. In contrast, the buccal mucosa of the rats treated with green tea extract maintained its structural integrity characterized by a normal underlying lamina propria and no inflammatory cell infiltration. The excised mucosa was also subjected to immunohistochemical evaluations using Ki-67 and Bcl-2 immunolabelling: Ki-67 is an antigen that provides information about the number of active cells and Bcl-2 is the most important gene encoding for protein controlling the mitochondrial membrane permeability and consequently apoptosis. As a result, the group receiving both Green tea extract and MTX maintained the normal level of Ki-67 expression, suggesting good cell proliferation activity, while in the MTX-treated group, a significant reduction in Ki-67 level was observed. Furthermore, green tea extract significantly increased the anti-apoptotic activity of Bcl-2 by upregulation of its expression. In conclusion, the antioxidant, antiangiogenic, and anti-apoptotic effects of the polyphenols-rich green tea extract might represent a valuable strategy to prevent chemotherapy-induced OM.

Over the past years, several studies attributed the well-known beneficial effects associated with grape juice or wine consumption to the relevant pool of flavonoids that they possess. These include catechins, epicatechin, Proanthocyanidins, and tannins. In this view, in 2017, Ahmed Saleh et al. [147] evaluated the effectiveness of a grape seed extract in the treatment of chemotherapy-induced OM. Forty-eight adult male rats were divided into four groups: (i) a negative control group receiving no treatment; (ii) a positive control group treated with Cetuximab by intraperitoneal injection (0.25 mg); (iii) a third group receiving simultaneously 0.25 mg of Cetuximab (intraperitoneal injection) and 20 mg/day of grape seed extract (oral administration); and (iv) a fourth group receiving 20 mg/day of grape seed extract orally one week ahead of Cetuximab administration. The experiment was carried out for 16 days and, afterwards, animals were sacrificed, and each tongue was analysed. As reported, the grape seed extract decreased inflammation both by modulating the inflammatory pathways and by reducing ROS levels. The dissected tongues were analysed by optical microscopy and scanning electron microscopy, choosing filiform papillae as a specimen as they are widely present on the dorsal surface of the tongue and very susceptible to drug cytotoxicity. Authors highlighted that Cetuximab was able to induce diffuse destruction of the tongue structure, resulting in increased susceptibility to bacterial infections. This pattern was completely reversed when the grape seed extract was administered. Additionally, significant differences have been highlighted in the third and fourth groups. Specifically, the administration of the grape seed extract with concomitant chemotherapy resulted in a reduction of tongue damage, showing only fewer colonies of microorganisms. In contrast, the pre-treatment with the extract gave the best results as this group was characterized by the lowest inflammation level and just sporadic atrophic areas detectable in the filiform papillae.

#### 5.6.2. Clinical Trials

Several flavonoids-rich plants have been used for centuries in traditional medicine to improve humans’ quality of life. To ensure that they may be particularly useful in maintaining and restoring the homeostasis of the oral tissues affected by mucositis, human clinical studies on human volunteer patients are needed in addition to the previously reported, preliminary, in vivo animal studies. This is necessary because therapeutic outcomes are often species-dependent, and thus animal studies need to be confirmed in humans. In this regard, just a few clinical trials are available to determine the effectiveness of flavonoids-rich plants in preventing and healing the chemo- and radiotherapy-induced OM. In 2013, Babaee et al. [139] evaluated the effectiveness of a *C. officinalis* flowers extract-loaded mouthwash gel to treat OM in patients with head and neck cancer undergoing telecobalt radiotherapy (200 cGy-5 days/week) alone or in combination with concurrent chemotherapy (5-FU or Cisplatin). Forty patients were recruited and equally divided into two groups treated with 5 mL of mouthwash in the form of a carboxymethyl cellulose-based oral gel containing 2% (*w*/*v*) of a *C. officinalis* flowers ethanolic extract twice a day or a placebo gel, for a total of 7 weeks. According to the authors’ investigation, by using the *C. officinalis* extract-loaded mouthwash both a reduction in the OM severity and a delayed OM onset were noticed when compared to the placebo group. This effect could be attributable to the antioxidant properties of flavonoids (e.g., Quercetin) and generally polyphenols abundantly present in this plant and responsible for the protective effect against mucosal injury due to radiotherapy. Furthermore, despite the presence of ethanol in the formulation could induce irritation, no serious side effects were observed during and after the treatment. However, as suggested by authors the use of a well-designed mucoadhesive formulation instead of a simple gel or the employ of higher doses of extract would be more effective. In 2015, Miranzadeh et al. [148] investigated the effectiveness of *A. millefolium* distillate against chemotherapy-induced OM. *A. millefolium* is a flavonoids-rich plant containing luteolin, kaempferol, and apigenin, which possess antioxidant and anti-inflammatory properties, thereby both acting against the key factors regulating the OM onset and contributing to the healing process of oral injured tissue [128].Fifty-six patients undergoing chemotherapy were randomly divided into experimental and placebo groups in similar blocks based on OM severity. The control group was treated with a routine mouthwash containing Lidocaine (1.4 g/L), Dexamethasone (0.224 g/L), and Sucralfate (35 g/L) in a Diphenhydramine commercial solution. In contrast, the experimental group received a mixture (50:50 *v*/*v*) of the routine mouthwash and the *A. millefolium* distillate (final concentration: 12 ppm). The administration protocol consisted of holding 15 mL of both mouthwashes for 3 min in the mouth, gargling, and then discarding. Patients could not wash their mouths or eat for one hour after administration and were advised to perform mouthwashing for a total of 14 days. The results showed a remarkable regeneration of the injured mucosa and a reduction in the OM severity in patients treated with the *A. millefolium* extract containing mouthwash compared to the placebo group. The latter, at the end of the experiment, showed a high percentage of patients with severe OM. The few here reported clinical trials still contribute to the main idea of using polyphenols-rich plant extract as a preventive strategy or adjuvant treatment in the management of both chemo- and radiotherapy-induced OM. The studies collected and reported are summarized in Table 2. 

## 6. Conclusions and Future Prospectives

Considering the high incidence of OSCC as well as chemo- and radiotherapy as the most common therapeutic approaches to fight against this highly malignant cancer type, finding novel strategies to minimize the treatments-related side effects, thus increasing as much as possible patients’ quality of life is a mandatory public health problem to be solved [5,6]. As reported, the most common adverse effect in patients undergoing both radio- and chemotherapy is OM, which is greatly exacerbated when these two treatment options need to be performed in combination. OM is a complex and multifactorial disease that adversely affects patients’ everyday lives, as it is characterized by pain, erythema, ulceration, and impairment of normal oral functions such as drinking, eating, talking, and, in extremely severe cases, even opening the mouth [5]. Its onset is characterized by a multifactorial etiopathogenesis primarily associated with the existence of a high inflammatory and pro-oxidant environment directly and/or indirectly created by both radiations and chemotherapeutics [20]. In this context, it should be useful the administration of natural antioxidant and anti-inflammatory compounds which can be characterized by significant effectiveness and reduced side effects. Among them, polyphenols could be particularly promising due to their wide range of biological actions, including relevant scavenger and wound healing properties, and the results are helpful both for OM prevention and treatment [33]. The papers here collected always report on the beneficial effects of several polyphenols alone or in combination (e.g., complex plant-derived extracts) against chemo- and radiotherapy-induced OM. The literature studies collected highlight the dual action of these powerful molecules which might act at different stages of OM development, thus being able to both delay OM onset and accelerate the complete tissue restoration, while also significantly reducing pain and functional impairment. Nevertheless, the actual clinical use of these promising compounds still remains a major challenge. Indeed, polyphenols generally possess disadvantageous physico-chemical properties, such as low water solubility and difficult handling due to high instability (light exposure, alkaline pH, and high temperature). These negative characteristics, together with a high susceptibility to both the gastrointestinal environment and the first-pass effect, severely compromise their bioavailability, especially when administered orally [51,149]. Over the past decades, researchers have then explored novel technological approaches to overcome these limitations. Several recent studies in the literature have investigated the possibility of efficiently embedding polyphenols into various delivery platforms in order to improve their solubility and stability and thereby increase their bioavailability [150,151,152,153]. Among the most promising delivery platforms, micro- and nanoparticulated delivery systems are the new frontier of drug delivery thanks to their ease of preparation, their ability to control drug release rate, as well as their versatility offered by a wide range of useful excipients (e.g., lipids, polymers, inorganic or hybrid materials). Several recent examples of successful loading of polyphenols into micro- and nano-delivery systems are easy to find. Dinesh Kumar et al. [154] entrapped QRC into biodegradable polycaprolactone-based nanoparticles providing a controlled release of the QRC amorphous form over 48 h. Similarly, Kazmi et al. [155] incorporated APG into polymer–lipid hybrid nanoparticles. Furthermore, Sulaiman et al. [156] have successfully produced gold nanoparticles containing HSP, while Safwat et al. [157] embedded in the same platform also EGCG. Additionally, also polysaccharides, such as chitosan, have been shown to effectively control the release rate of polyphenols [158]. All these formulations are also versatile in terms of administrability as they can be administered as nanoparticles dispersion/powder or further incorporated into different matrices, including bioerodible sponges [159], hydrogels [160], or scaffolds [161]. As claimed in this paper, there are two other main factors to be considered: (i) as micro- and nano-particulate drug delivery systems can enhance polyphenols bioavailability and active cells interaction, several in vitro, in vivo and clinical studies will be required to evaluate the right dose to be administered in order to achieve the strongest therapeutic outcomes without any risk [123]; (ii) the effectiveness of polyphenols in the management of OM has been in vivo and clinically proven by administering the actives either orally or topically. In both cases, very few advanced technological formulations have been tested (generally conventional mouthwashes, solutions/dispersions, or gels, and the only reported exception is related to SinaCurcumin^®^ capsules) and thus the therapeutic outcomes might be limited due to numerous factors such as gastrointestinal degradation, first-pass effect metabolism, swallowing and taste-enhanced salivary turnover. Consequently, the development of innovative and safe mucoadhesive formulations for topical administration to the injured mucosae could maximize the preventive and healing effects at extremely low doses while also minimizing the active entry into the bloodstream and consequently any systemic side effects. The buccal administration of polyphenols is currently under the spotlight, as it could be the smartest strategy to treat several oral pathologies besides OM, such as periodontitis, bacterial infections, and biofilms formations, as well as MRONJ due to anticancer therapy, oral lichen planus, etc. [162,163,164,165]. In this context, employing ad hoc designed mucoadhesive formulations allows enhanced retention of the drug on the surface of application and action, together with protection from degradation and leaching due to the presence of saliva, as well as controlled drug release pattern, ease of application, and generally high patient compliance [150,166].

## Figures and Tables

**Table 1:** Effects of curcuminoids in prevention and treatment of chemo- and radiotherapy-induced OM: administration modalities, used models, and general outcomes.

Compound/Formulation, Route of Administration, Dose, and Regimen	Type of Study	Animals/ Participants	Model	Outcomes	References
CUR (20 mg/mL) mucoadhesive gel; topical administration of 0.5 g twice a day for 2 weeks, compared to control, placebo, and *C. recutita* fluid extract	In vivo animal study	Golden Syrian hamsters	Chemotherapy-induced OM (5-FU)	Reduced angiogenesis, vascularization, and TGF-β1 labelling as well as rapid healing and reepithelization	Schmidt et al., 2019 [63]
CUR 50 or 100 μg/mL hydroalcoholic solution; topical administration of 0.25 mL/day, compared to control, placebo, QRC, and peptides, for 20 days.	In vivo animal study	Golden Syrian hamsters	Radiotherapy-induced OM (single dose: 40 Gy)	Massive reduction of OM severity in a dose-dependent manner	Dvoretskiy et al., 2022 [64]
CUR (0.1% *w*/*v*) mouthwash, topical administration of 10 mL 3 times/day for 6 weeks, compared to a commercial benzydamine mouthwash (0.15% *w*/*v*, COOLORA™)	Triple-blind clinical trial	Adult patients with OSCC	Radiotherapy-induced OM (60–70 Gy)	Delayed onset of OM by two weeks in half of the patients and no one displayed severe OM	Shah et al., 2020 [66]
CUR (7.5 mg in 10 mL) solution; topical administration once a week for 1 month in combination with photodynamic therapy (450 nm blue LED) compared to low-level laser irradiation (600 nm) and control	Clinical trial	Adult patients	Chemo- and radiotherapy-induced OM	Reduction of *Candida albicans* infection, together with less pain and lower OM scores	de Cássia Dias Viana Andrade et al., 2022 [67]
CUR gel (0.5% *w*/*v*); topical administration 4 times/day for 2 weeks compared to Mucosamin^®^ oral spray (0.2% *w*/*v*) and Chlorhexidine mouthwash (0.5% *w*/*v*)	Double-blind clinical trial	Adult patients	Chemotherapy-induced OM	Reduction in OM severity (WHO and OMAS scores) and faster lesion restoration (within 4 days) compared with the other formulations	Fardad et al., 2022 [68]
Capsules containing curcuminoids and essential oil of turmeric (0,5 g); per os twice or 3 times/day (1 or 1.5 g/die) for 6 weeks, compared to placebo	Double-blind clinical trial	Adult patients	Chemotherapy-induced OM	Reduction of OM symptoms and severity in a dose-dependent manner	Soni et al., 2022 [69]
SinaCurcumin^®^ capsules (80 mg CUR) per os once a day for 6 weeks, compared to placebo	Double-blind clinical trial	Adult patients	Radiotherapy-induced OM (50 Gy)	Delay in the OM onset (one week). Reduction in OM severity and lower weight loss	Delavarian et al., 2019 [72]
SinaCurcumin^®^ capsules (80 mg CUR) per os twice a day for 7 weeks, compared to placebo	Clinical trial	Adult patients	Chemo- and/or radiotherapy-induced OM (30–50 mg Cisplatine or 640–750 mg 5-FU and/or 60–70 Gy)	Reduction of pain and OM signs, severity, and progression. The beneficial effects enhanced for patients receiving only chemotherapy.	Kia et al., 2021 [73]
SinaCurcumin^®^ capsules (40 mg/die) per os 3 times/day, for 21 days compared to CUR mouthwash (0.1% *w*/*v*) topical administration 3 times/day, and placebo	Randomized clinical trial	Adult patients	Radiotherapy-induced OM	Complete ulcer restoration as well as reduction of OM severity, signs, and symptoms. No statistical difference in the two CUR groups was observed.	Ramezani et al., 2023 [74]

**Table 2:** Summarizes the information of the papers selected and above discussed.

Tested Substance(s)	Dose, Regimen, and Route of Administration	Type of Study	Animals/ Participants	Model	Outcomes	References
α-glucosyl hesperidin (α-G-HSP)	α-G-HSP 1 mg/mL ad libitum, from 5 days before 5-FU treatment until 16 days. Oral administration	In vivo animal study	Golden Syrian hamsters	Chemotherapy-induced OM (5-FU 60 mg/kg by intraperitoneal injection)	Preventive effect leading to smaller ulceration areas due to inhibition of ROS and lipid peroxidase	Yoshino et al., 2016 [92]
(−)-Epicatechin (EC)	100 μL/dose of a 2 mM EC solution three times a day compared to control for 23 days. Oral administration.	In vivo animal study	Sprague-Dawley rats	Radiotherapy-induced OM (30 Gy)	Inhibition of radiation-induced apoptosis, reduction of ROS, and NOX-e protein production. Improvement of animals’ viability.	Shin Y.S. et al., 2013 [100]
Epigallocatechin-3-gallate (EGCG)	15 mL of EGCG-loaded mouthwash at increasing doses (from 440 to 2200 μM), 3 times/day for 8 weeks. Topical administration.	Clinical trial	Adult patients	Radio- and sometimes concurrent chemotherapy-induced OM (66–72 Gy)	Each dose was able to reduce pain and OM severity, resulting in no patients showing severe OM. The chosen recommended dose for a further phase II clinical trial is equal to 1760 μM.	Zhu et al., 2020 [108]
Apigenin (APG)	40 mg/Kg of APG potassium salt solution by gavage compared to dexamethasone (1 mg/Kg) and control, for 14 days. Oral administration.	In vivo animal study	Golden Syrian hamsters	Chemotherapy-induced OM (5-FU 60 mg/kg by intraperitoneal injection)	Significant reduction of OM severity and acceleration of the normal wound-healing process	Molina Prats et al., 2017 [113]
Quercetin (QRC)	0.25 mL/day of QRC in DMSO solutions 50 or 100 μg/mL compared to control, placebo, CUR and peptides, for 20 days. Topical administration.	In vivo animal study	Golden Syrian hamsters	Radiotherapy-induced OM (single dose: 40 Gy)	Both doses determined a strong reduction of OM severity but only the QRC 100 treatment was considered effective	Dvoretskiy et al., 2022 [64]
Quercetin (QRC)	300 mg/Kg/day of QRC from one week before radiotherapy and until 8 days after radiotherapy. Oral administration.	In vivo animal study	C57BL/6 mice	Radiotherapy-induced OM (single dose—25 Gy or fractionated dose—8 Gy/day for 3 days)	Reduction of lesions’ size and mucosal damage. Protection against the radiation-induced reduction of Ki-67 and PCNA. Inhibition of P-21, P-16, and expression of pro-inflammatory cytokines.	Zhang J. et al., 2021 [122]
Quercetin (QRC)	5 mg/kg/day of free QRC or QRC-loaded nanoemulsion as a pre-treatment or post-treatment, for 13 days. Oral administration.	In vivo animal study	Albino mice	Chemotherapy-induced OM (300 mg/kg)	Enhanced general health. Preservation of the mucosal integrity with reduction of inflammatory cell infiltration. Reduction of MDA and increase in SOD and CAT expressions.	Lotfi et al., 2021 [123]
Quercetin (QRC)	QRC hydrate-loaded capsules (250 mg/dose) compared to placebo, twice a day for 4 weeks. Oral administration.	Randomized placebo-controlled double-blind clinical trial	Adult patients	Chemotherapy-induced OM (predominately Cytarabine or Daunorubicin, but also Vincristine, Cyclophosphamide, Plasil, Idarubicin, Fludarabine, Adriamycin and L-asparaginase)	Reduction of OM incidence (from 60% to 30%)	Kooshyar et al., 2017 [124]
Hydro-alcoholic *H. perforatum* extract and *P. atlantica* oil extract	Hydro-alcoholic *H. perforatum* extract-loaded gel (10% *w*/*v*) or *P. atlantica* oil extract-loaded gel (10% *w*/*v*) or combination thereof for 18 days. Topical administration.	In vivo animal study	Golden Syrian hamsters	Chemotherapy-induced OM (60 mg/kg by intraperitoneal injections)	Lower micro- and macroscopic OM scores. Enhanced healing and reduced levels of oxidative stress markers. These effects are enhanced when the two extracts are administered in combination.	Farrokhi et al., 2017 [131]
Hydro-alcoholic *C. officinalis* extract	Hydro-alcoholic *C. officinalis* extract loaded mucoadhesive gel (5 or 10% *w*/*v*) once a day, compared to placebo and control for 17 days. Topical administration.	In vivo animal study	Golden Syrian hamsters	Chemotherapy-induced OM (60 mg/kg by intraperitoneal injections)	Reduction of haemorrhage, inflammatory cell infiltration, and ulceration. Dose-dependent wound healing properties.	Tanideh et al., 2013 [136]
Standardized bilberry extract containing 36% (*w*/*v*) of glycosylated Anthocyanins (ANTs)	45 mg/mL (corresponding to 300 mg/kg) of aqueous solution containing the bilberry extract once a day by gavage for 7 days before chemotherapy. Oral administration.	In vivo animal study	Golden Syrian hamsters	Chemotherapy-induced OM (60 mg/kg by intraperitoneal injections)	The pre-treatment with the bilberry extract determined the enhancement of mice’s general health, together with a reduction of OM severity and signs	Davarmanesh et al., 2013 [142]
ANTs-rich *Oryza sativa* L. extract	500 or 1000 mg/Kg of *Oryza sativa* L. extract once a day by gavage for 29 days compared to control. Oral administration.	In vivo animal study	Wistar rats	Chemotherapy-induced OM (60 mg/kg by intraperitoneal injections)	Dose-dependent wound healing properties due to reduction of p50, p65, NF-κB and HMGB1 markers	Tancharoen et al., 2018 [143]
Green tea extract	40 mg/Kg of green tea extract by gavage once a day, compared to control for 5 days. Oral administration.	In vivo animal study	Albino rats	Chemotherapy-induced OM (MTX from 10 to 80 mg/kg by intraperitoneal injection)	No buccal mucosa damage and reduction of inflammatory cell infiltration. Normal Ki-67 expression together with up-regulation of Bcl-2.	Al-Refai et al., 2014 [129]
Grape seed extract	20 mg/day of grape seed extract as a pre-treatment (from one week before chemotherapy) or in concomitance with chemotherapy, for 16 days, compared to control. Oral administration.	In vivo animal study	Rats	Chemotherapy-induced OM (0.25 mg of cetuximab by intraperitoneal injection)	Reduction of tongue damage, ROS levels, and susceptibility to bacterial infection. By the pre-treatment, a limited inflammatory stage and lower atrophic areas in the mucosa were observed.	Amhed Saleh et al., 2017 [147]
*C. officinalis* flowers ethanolic extract	5 mL of a *C. officinalis* extract-loaded mouthwash gel (2% *w*/*v*) twice a day compared to placebo for 7 weeks. Topical administration.	Clinical trial	Adult patients with head and neck cancer	OM induced by radiotherapy (200 cGy-5 days/week) alone or in combination with chemotherapy (5-FU or Cisplatin)	Delayed OM onset and reduction of OM severity of OM Despite the presence of ethanol or irritation, no other serious side effects were observed	Babaee et al., 2013 [139]
*A. millefolium* distillate	15 mL of *A. millefolium* distillate-loaded mouthwash (12 ppm) by gargling for 3 min daily compared to placebo for 14 days. Topical administration.	Clinical trial	Adult patients	Chemotherapy-induced OM	Remarkable regeneration of the injured mucosa and reduction in OM severity	Miranzadeh et al., 2015 [148]
